# Policies to mitigate health inequity: a comparison of Israel and 24 developing countries

**DOI:** 10.1186/s13584-016-0105-4

**Published:** 2016-11-10

**Authors:** Daniel Cotlear

**Affiliations:** Universal Health Coverage Study Series, World Bank Group, The World Bank, 1818 H St. NW, Washington, DC 20433 USA

## Abstract

Efforts to mitigate health inequity are at the heart of health policy in Israel and in many developing countries seeking to advance toward universal health coverage. This commentary uses the conceptual framework and the description of health policy interventions presented in a recent IJHPR article to compare policies implemented by Israel’s Ministry of Health during 2011–2014 with policies under implementation in 24 developing countries, and identifies key differences and similarities. It also identifies three areas of policy where Israel seems to have strong capacities that are in high demand in developing countries. Identifying these areas of policy could help design a menu for Israeli technical assistance in health policy.

## Background

In a recent IJHPR article, Tuvia Horev and Shlomit Avni developed a conceptual framework which they use to describe the efforts of Israel’s Ministry of Health to develop a policy to mitigate health inequity [1]. In this commentary we use the Horev-Avni framework (hereafter H-A framework) to compare the pro-equity policy interventions applied by Israel during 2011–2014 with health policy used by 24 developing countries committed to advancing universal health coverage. The paper begins with a comparison of the H-A framework with another framework that has gained great currency in recent years among developing countries, the universal health coverage (UHC) framework [2]. It concludes by identifying a “menu” of interventions where Israel may offer lessons in capacity building that may be useful for the many developing countries that are today pursuing UHC and the greater health equity it promises.

The two frameworks share similar boundaries which makes their comparison possible. In their study, Horev and Avni define the boundaries of their analysis as those efforts to mitigate health inequity which are under the influence of the National Health Authority. They recognize the evidence presented by the literature related to the social determinants of health disparities and support the 2011 Rio Declaration expressing the WHO member states’ commitment to fighting health inequity by addressing the social determinants of health [3]. While recognizing the broader multi-sectoral effort required to take action on the social determinants and to fight social and economic inequity (“health equity in all policies and activities”), they convincingly argue that it is also important to have a strategy dealing more narrowly with the health inequities that can be influenced by the National Health Authority – in this case Israel’s Ministry of Health (MoH). The universal health coverage concept has gained much currency in recent years as a paradigm for the advancement of health equity. This concept was popularized by the WHO’s World Health Report of 2010 using what is now often referred to as the UHC cube [2]. The UHC cube diagrammatically illustrates the idea that UHC has three dimensions: The first is the population covered, the second is the benefits covered and the third is the financial risk protection covered. Equity is a core principle of UHC; the importance of equity is shown by the emphasis it receives in the proposed monitoring instruments that have been developed to track progress towards UHC [4]. In countries where population coverage is not universal, making it universal is perceived as a first step. A UHC “movement” has rapidly expanded in developing countries reaching the point where UHC is now one of targets for the Sustainable Development Goals. A recent survey of 46 African countries found that 42 of them are officially committed to achieving UHC [5].

UHC occupies a similar conceptual space as the H-A framework – by recognizing that much of the inequities result from the social determinants of health, but focusing on policies that are under the purview of national health sector authorities. The concept of UHC was defined by the 58^th^ session of the World Health Assembly in 2005 as “access to key promotive, preventive, curative and rehabilitative health interventions for all at an affordable cost”.

## A comparison of pro-equity policy interventions using the H-A framework

The H-A paper develops a framework and uses it to organize the description of a wide set of policy interventions implemented by Israel during 2011–2014. Their framework is based on four “focal points of intervention”: (i) accessibility, (ii) availability, (iii) quality and efficiency and (iv) fairness. In the rest of this commentary, we adopt the same framework to summarize the key policy interventions used by 24 developing countries who have in place a UHC reform program – defined as reform programs aiming to expand health coverage in ways that are inclusive of the poor. The comparison is summarized in Table 1. The source of data for the 24 developing countries is the Universal Health Coverage Study Series (http://www.worldbank.org/en/topic/health/publication/universal-health-coverage-study-series). This series includes 24 country reports of low- and middle-income countries prepared using the “UNICO” data collection instrument. The instrument includes questions related to the three dimensions of the UHC cube described above; additionally, the UNICO instrument also includes questions about availability of health providers and about accountability. A synthesis of the 24 country reports and the data collection instrument was published as a book, which identifies the frequency with which each type of policy is used within the 24 countries [7].

**Table 1:** A comparison of pro-equity policy interventions in Israel and in 24 developing countries using the Horev-Avni framework

Type of Intervention classified by the AH model’s “Focal points of intervention”	Policy interventions implemented by Israel”s MoH during 2011–2014 to promote health equity [1]	Policy interventions frequently used in developing countries implementing UHC reforms [7]
Accessibility		
Overcoming economic barriers by improving affordability	Relief in copayments for targeted users; inclusion of additional benefits (e.g., pediatric dental health) into statutory benefits package for targeted users	New instruments are implemented to reduce user fees at the point of service. All UHC programs include fully subsidized programs for the poor. For the non-poor, some countries use contributory and others use non-contributory schemes. Most developing countries are expanding the statutory BP (benefits package).
Overcoming economic barriers by developing payment system incentives	Prospective payments to incentivize HMOs to invest in the periphery; retrospective payments conditioned on specific targets being met in the periphery; auditing tools developed to enforce compliance with equity targets and cultural competence	Most countries are experimenting with new forms of contracting and paying health care providers. Many countries are experimenting with incremental autonomy for public hospitals. Half of governments are now contracting with private sector health care providers.
Overcoming cultural barriers	Setting of norms in cultural and linguistic access to care (e.g., appointment of a cultural official in each establishment); introduced telephone translation services; introduced multi-media compulsory cultural training system for health care staff.	Most countries are in a process of decentralization; in many cases this is expected to strengthen ethnic representation. Some countries are considering inclusion of traditional medicine in statutory benefits packages, but this is not common.
Overcoming information barriers	Information on health rights for citizens and executives describing specific entitlements of vulnerable or disadvantaged populations. Synthesized, translated into Arabic and Russian, backed up by a call center.	Know-your-rights communications are common, including greater transparency about user fees and services in the statutory BP. Also, some countries are experimenting with “charters of patient rights”.
Availability		
Improving the distribution of staff	Expanding training capacities in peripheral areas (e.g., new medical school in Galilee); the new collective agreement with the Israel Medical Association includes significant incentives linked to service in peripheral areas; a national ceiling in hiring of physicians was lifted for service in the periphery; targets set to train health care personnel from specific ethnic groups.	Developing countries typically have huge disparities in the geographic allocation of human resources for health. While urban areas have a relative abundance of health professionals, rural areas and poor urban areas tend to have very few. Most of the 24 developing countries have policies to attract professionals to underprovided areas, but they do not seem to work for various reasons, including the lack of national ceilings (see text).
Improving the distribution of physical infrastructure	Ceilings (in hospital beds, MRI, line accelerator) were lifted by the Ministry of Finance with priority assigned to periphery; special budget created to build up health care infrastructure in the periphery. Developed and imposed standards of waiting times and distance to medical service.	Most developing countries have policies to expand investments in physical infrastructure. They often do not have minimum standards, so often the new investments are not sufficient to provide access to high-in-demand interventions included in the BP. Also, few countries have ceilings so the investments are not made within a priority framework.
Incentivizing use of technologies	Growing use of tele-medicine to improve the quality of services provided in the periphery.	There is high interest in, but very incipient use of tele-medicine
Quality and Efficiency		
Capacity building within the MoH	Establishing a dedicated national research unit on health inequality funded by the MoH	There is much interest in the concept, but few documented cases of units linked to the MoH or another National Health Authority dedicated to monitoring or researching progress towards UHC or health equity.
	Publication of an annual report on health inequality	There is no documented commitment to an annual public report on coverage or on health inequality.
Capacity building in the health policy community	Significant investments in quality indicators Analysis of data disparities in quality indicators, based on a well-developed national system for monitoring clinical quality	There is substantial availability of household survey data and widespread efforts of analysis from household surveys. There is increasing availability, but very limited use, of administrative data. The systems of information related to clinical quality are very weak.
	Establishing an annual national conference on health inequity	There is one documented effort (in the Philippines) to hold annual national conference with attendance of policy makers on topics related to UHC or health equity.
Fairness		
Funding	Progressivity in funding	Multiple efforts are under way to measure and monitor the progressivity in the funding of public health care
Representation	Adequate representation of diverse cultural backgrounds	Awareness of the need for the representation of diverse cultural backgrounds is increasing; there are many ongoing efforts to increase diversity in representation, but often these are linked to the process of decentralization rather than the process of health reform.
Community empowerment	Community empowerment such as public participation in policy formulation and interventions with Civil Society Organizations related to underserved groups and Ethiopian Israelis	There are many efforts of community empowerment in different areas.

### Accessibility

There are many similarities in this area as countries aim to overcome the economic, cultural and informational barriers.

Regarding economic barriers, Israel has sought to provide relief in copayments and an increase in the statutory BP for targeted users. Developing countries have made the reduction of payments at the point of service one of the main areas of intervention. All developing countries implementing UHC reform programs have a policy of eliminating user fees for their populations under the poverty line. Most countries cover the formal sector populations with mandatory contributory health insurance schemes. Where developing countries take different routes is in relation to the coverage of the non-poor self-employed – some countries have chosen to tax finance them, while others attempt to develop the capacity to get them to contribute toward the cost of the scheme.

Israel is using payment systems, both prospective and retrospective, to make it more attractive for HMOs to serve peripheral populations. It also uses a well-developed system of risk adjustors as part of the payment system. Most developing countries are starting to experiment with modern payment systems and moving away from the use of historic budgets. As they begin to develop capacities in this area, they often face a lack of off-the-shelf solutions. In many developing countries there are efforts to find objective ways, such as risk adjustors, to assign resources in relation to relative poverty or (especially in countries with rapidly aging populations) age groups. Developing countries also need to overcome the existence of rigid rules of financial management that limit the autonomy and flexibility of public sector health care managers.

In relation to the cultural barrier, the authors describe a very practical set of measures taken in Israel. Ethnic and cultural factors are very important in developing countries; however, there is no systematic research regarding the treatment of this barrier by developing countries. One may speculate that, in developing countries, the main response to this barrier is decentralization – a recent study of 46 African countries found that a whopping 42 countries are in the middle of a process of decentralization of health care.

### Availability – as it relates to the geographic distribution of resources

The Horev and Avni framework emphasizes equity in the availability of services by focusing on the geographic distribution of health care resources. This is a very significant problem in developing countries. The inequity in the distribution of human resources for health is perceived by many as one of the key barriers to UHC and has led to an entire body of literature analyzing this [6]. The vast majority of the 24 countries studied by UNICO have policies in place to attract health professionals to rural areas by providing incentives (such as bonus payments or points towards sought after residency programs) and penalties (such as a rural stage to graduate or to be hired as a civil servant). The evidence about the effectiveness of these policies suggest that these policies may not be having a big impact in reducing the urban to rural gap.

The Israeli experience described by Horev and Avni stands in contrast to the experience of the 24 developing countries in one important way. While in both settings there exist efforts to increase resources in poor regions, the policy in Israel additionally sets an array of national ceilings which ensure that the relative attraction of the poor areas improves. Ceilings are described for medical school class sizes, hiring of professionals, installations of MRIs and of other high-end technology. By contrast, we have no documented evidence of the existence of any such ceilings in urban settings in developing countries. For this reason, government efforts to improve the attraction of rural or poor areas for health workers often fail because salaries, training and high tech equipment grow faster in urban areas propelled by richer local governments and by the fast-growing (and mostly unregulated) private sector.

### Quality and efficiency

The Horev and Avni framework includes a focal point they designate as “quality and efficiency” that includes several interventions in the area of Governance and Monitoring and Evaluation. This is an area that has attracted many interventions both in Israel and in the 24 developing countries. In both there is a growing availability of, and capacity to use, household survey information for the analysis of equity. However, in addition to this, Israel has developed a capacity to use computerized data on clinical quality to monitor equity and has introduced very significant institutional innovations which may sustain attention on equity issues. These innovations include the establishment of a unit in the MoH specialized in the monitoring of equity and the official promise to publish a yearly report monitoring health equity. In addition, as an effort designed to promote research on equity within the broader academic and civil society communities – an annual conference with the presence of policy makers has been instituted. By contrast, a significant finding of *Going Universal* is that in developing countries, while there has been a significant increase in data availability, there are still few efforts of regular reporting of monitoring indicators to the public. While many countries are considering the possibility of establishing a specialized Health Equity or UHC unit under the MoH, to date very few have succeeded at doing so.

## Conclusions

The discussion above suggests that the framework developed by Horev and Avni is useful for conceptualizing and comparing health equity interventions that fall under the domain of a National Health Authority such as a Ministry of Health. The comparison of the Israeli experience during 2011–2014 with the experience of 24 developing countries advancing UHC reforms as summarized in *Going Universal* leads to the following conclusions:

In developing countries a major effort in the quest of health equity is to expand coverage. Israel has achieved statutory coverage for all its population and developed a more specific set of health equity priorities for implementation during 2011–2014. Despite this difference, the developing countries and Israel are intervening in similar “focal points of intervention”, even if the specific interventions are sometimes different. This commentary identified three areas where Israel has developed strong institutional capacities that are now sought by developing countries and could serve as a guide for future efforts of international technical assistance. Specifically:

- Israel has an advanced capacity for the development and implementation of payment systems to health providers; notably, the Israeli system requires the explicit use of risk adjustment. Increasingly developing countries are seeking to modernize their payment systems and are increasingly identifying the need to develop explicit risk adjustment mechanisms, especially for the poor and for the elderly.
- Israel makes ample use of national ceilings to regulate the development of the supply of health care provision in a pro-equity direction. In developing countries ceilings to enforce regulation are rarely utilized and many efforts to prioritize certain regions or populations often fail because there is no control over high tech investments or the training and hiring health professionals in the most attractive and richer locations.
- Israel has expanded the space for policy dialogue between the government, civil society and the academic community – the annual reporting on progress in health equity and the now well established annual conference on health inequity are important examples of this. So is the creation of a unit to monitor and advise on health equity policy. These are areas where developing countries tend to be relatively weak. There is now much greater availability of household survey information, but there is still little data available about clinical quality or even administrative data on use of services (or reimbursement for services provided to different populations). Many countries are exploring the creation of a “UHC unit”, but this is still uncommon. Even more uncommon, is the existence of public funding to promote health policy research by the local academic community and efforts to facilitate the dialogue of the academic community with policy makers.

## Abbreviations

BP: Benefits package
H-A: Horev-Avni
MOH: Ministry of Health
UHC: Universal health coverage
UNICO: Universal coverage study series
